# Temperature gradient sensing mechanism using liquid crystal droplets with 0.1-mK-level detection accuracy and high spatial resolution

**DOI:** 10.1038/s41598-022-18008-y

**Published:** 2022-08-12

**Authors:** Shinji Bono, Satoshi Konishi

**Affiliations:** 1 Department of Mechanical Engineering, College of Science and Engineering, Ritsumeikan University, Kusatsu, 525-8577 Japan; 2 Graduate Course of Science and Engineering, Ritsumeikan University, Kusatsu, 525-8577 Japan; 3 Ritsumeikan Advanced Research Academy, Kyoto, 604-8520 Japan; 4 Ritsumeikan Global Innovation Research Organization, Ritsumeikan University, Kyoto, 604-8520 Japan

**Keywords:** Liquid crystals, Sensors and biosensors

## Abstract

We proposed the detection mechanism of the micro-levels of temperature gradient in a micro-electromechanical system using the unidirectional rotation of cholesteric-liquid crystal (Ch-LC) droplets. Ch-LC droplets in the presence of an isotropic phase subjected to a heat flux rotate with a speed proportional to the magnitude of the temperature gradient. We further quantified the temperature gradient-to-torque conversion efficiency to apply the thermomechanical cross-correlation to the detection of temperature gradient. Then, we observed the rotational behavior of Ch-LC droplets after introducing them onto model devices containing patterned Au thin-film electrodes. Direct electric current applied to these Au electrodes results in unidirectional rotation of the Ch-LC droplets in response to heat flux generated from the Au electrodes. By evaluating the possible temperature gradient detection resolution using Ch-LC droplet rotation, we show that Ch-LC droplets can achieve both high spatial resolution (~ 10 µm) and high detection accuracy (~ 0.1 mK/µm).

## Introduction

Electrical and chemical energy consumption must be accompanied by the dissipation of heat^1,2^. In a micro-electromechanical system (MEMS), for example, the electric power in wires, sensors, and actuators is dissipated as heat, whereas chemical reactions in biochemical systems also involve the partial conversion of chemical energy into heat. The heat flux distribution in MEMSs may provide particularly useful information to better understand the local electric properties of MEMS devices, such as identifying micro-defects^3^. The development of MEMS techniques has resulted in micrometer-sized sensors and actuators integrated into millimeter-scale devices^4,5^. The high-resolution characterization of heat flux distributions in MEMS devices plays a crucial role in this effort.

Cross-correlation is a principle commonly used to detect temperature gradient which is proportional to heat flux, a well-known example of which is Seebeck elements that use the cross-correlation between heat flux and electromotive force that enables temperature gradient in a system to be detected as electric potential^6–8^. The conversion efficiency from temperature gradient to electric potential is called the Seebeck coefficient, which is the ratio of output voltage to the temperature gradient in steady-state system. The output voltage from Seebeck elements is proportional to both the size of the device and the Seebeck coefficient^9^. Thus, high temperature gradient detection accuracy using devices composed of identical material requires a step up in device size, which often involves a reduction in spatial resolution. Therefore, improving the detection accuracy of temperature gradient while maintaining a high spatial resolution presents a significant challenge.

Recent studies have reported the thermomechanical cross-correlation between heat flux and torque in chiral liquid crystals (LCs), starting with that of O. Lehmann. This was the first study to document the heat-driven rotation of cholesteric (Ch)-LCs, and is therefore known as the Lehmann effect^10^. The rotational behavior can be qualitatively understood in the context of Leslie’s phenomenological theory^11^. The heat-driven rotation of the Ch-LC droplets dispersed in a corresponding isotropic (Iso) phase is a well-known example of such behavior^12–16^. Ch-LC droplets subjected to a uniform heat flux rotate unidirectionally, with a rotational direction and speed that depend on the Ch-LC chirality and the heat flux magnitude, respectively^17^. Note that the high conversion efficiency of the Ch-LC thermomechanical cross-correlation results because the droplets can directly convert heat flux into torque.

Previous work has focused on the relationship between the rotational behavior and the molecular director configuration of Ch-LC droplets, which strongly depends on the chirality of Ch-LCs, the size of droplets, and the boundary conditions^16–19^. Ch-LC droplets have a simple director configuration with a single twist (ST) orientational structure, in which the long axis of the molecular continuously twists along a helical axis^20^. To obtain ST Ch-LC droplets, the diameter of the droplets must be shorter than the pitch of the helix of the director configuration, where the pitch is inversely proportional to the concentration of chiral dopants. The heat-driven rotational behavior has also been investigated, for example, in terms of heat flux applied perpendicular to the helical axis. In this case, the ST Ch-LC droplets rotate with an angular velocity proportional to *R*^−2^∇*T*, where *R* and *T* are the radii of the Ch-LC droplets and temperature, respectively^13^. In addition to ST Ch-LC droplets, studies on molecules with other director configurations have reported that smaller Ch-LC droplets rotate faster, regardless of the director configuration. This is attributed to friction between the substrate and the Ch-LC droplets, which decreases with increasing size of the Ch-LC droplets^16,21^. This provides a framework for understanding how small Ch-LC droplets can sense heat flux with high accuracy.

As LC materials exhibit fluidity, one advantage of working with soft LC materials is that they can be inserted into spaces with confined or bending geometries^22–25^. In addition, heat flux detection using Ch-LC droplets is a promising candidate for applications involving not only rigid systems but also flexible microdevices^26^. We can calibrate the effect of the introduction of LC on a thermal property such as temperature gradient quantitatively by multiplying the ratio of thermal conductivities.

Previous work has focused primarily on the rotational behavior of Ch-LC droplets in the presence of uniform temperature gradient (heat flux). In this paper, we investigated the response of heat-driven rotation of Ch-LC droplets detection to a spatially heterogeneous temperature gradient, and then sought to determine the detection accuracy and spatial resolution. First, to quantify the conversion efficiency from temperature gradient to rotation, we observed the rotational behavior of Ch-LC droplets subjected to a uniform external temperature gradient. Next, we patterned the Au thin-film electrode using MEMS techniques and generated a local micro-heat flux by applying a direct current to the Au thin-film electrode. Then, after introducing Ch-LC droplets onto the Au thin-film electrode, we measured their distribution of angular momentum. Based on the conversion efficiency, we estimated the temperature gradient distribution from the angular momentum distribution of Ch-LC droplets for the first time. Then, we determined the spatial resolution and detection accuracy of the temperature gradient and proposed a novel principle of temperature gradient detection using Ch-LC droplets with high spatial resolution and high detection accuracy.

## Results

### Evaluation of temperature gradient to rotation conversion efficiency

**Figure 1:**
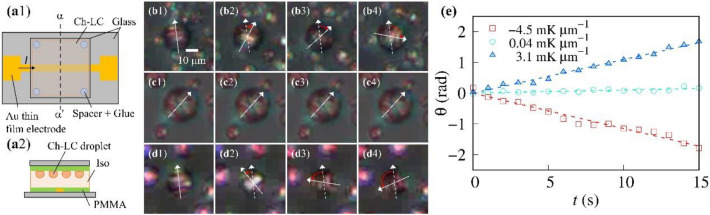
Ch-LC droplets subjected to a uniform temperature gradient. (**a1**) A schematic top-view image of the LC cell. We patterned an Au thin-film electrode on the lower glass substrate and introduced Ch-LCs between the upper and lower substrates. The spacing of the glass substrates was adjusted to 40 µm by bead spacers. (**a2**) Cross-section view (α-α′) of the LC cell. We used polymethyl methacrylate (PMMA) as an alignment film in the Ch-LC sample. Polarized microscopic images of Ch-LC droplets are shown for uniform temperature gradient values of (**b**) ∇*T* =  − 4.5 mK/µm (Supplementary movie 1), (**c**) ∇*T* = 0.01 mK/µm (Supplementary movie 2), and (**d**) ∇*T* = 3.1 mK/µm (Supplementary movie 3). White arrows and white dashed arrows indicate the direction of the helical axis of the director configuration and the initial direction of the axis, respectively. Snapshots were collected every 5 s. (**e**) The time evolution of θ. The dashed lines are best-fit lines obtained using linear functions.

We applied a uniform temperature gradient to Ch-LC droplets to measure the angular velocity of the rotating droplets and evaluated the efficiency of temperature gradient conversion to rotation. Ch-LC samples were sandwiched between two glass substrates with a sample thickness set at 40 µm. We patterned a 200-nm-thick Au thin-film electrode on the lower glass substrate and fabricated model MEMS devices as shown in Fig. 1a. Both ends of the electrode were connected to the DC power supply via an electrical resistor. First, the temperatures of the upper and lower glass substrates were varied independently using a homemade temperature controller. We can regard the temperature gradient through the model MEMS device as uniform because the thickness of the model MEMS device (> 2 mm) is larger than that of LC (~ 40 µm) enough. To estimate a uniform heat flux along the temperature gradient, we should multiply d*T*/d*z* by the heat conductivity of the device material (~ 0.1 W/K m). Then, we made polarized microscopic observations of the Ch-LC droplets in the presence of a uniform temperature gradient without applying current to the Au thin-film electrode. We defined the positive temperature gradient direction as upward.

Figure 1b and Supplementary movie 1 display polarized microscopic images of a Ch-LC droplet under uniform temperature gradient (d*T*/d*z* =  − 4.5 mK/µm, where we set the *z*-axis perpendicular to substrates). The gap in the striped texture of the Ch-LC droplet resulting from its twisted director configuration corresponds to the half-pitch of the helix (~ 10 µm). The helical axis of the director configuration is perpendicular to the striped textures. Under uniform temperature gradient conditions, the striped texture of the Ch-LC droplets rotates unidirectionally with a constant angular velocity in the clockwise direction independently of the droplet position. We confirmed that the chirality inversion of the Ch-LC sample causes the rotational direction to reverse, which is consistent with previous reports on chiral inversion characteristics and cross-correlation^17,27^.

Figure 1c,d show polarized microscopic images of Ch-LC droplets under uniform temperature gradient conditions of d*T*/d*z* = 0.04 (Supplementary movie 2) and 3.1 mK/µm (Supplementary movie 3), respectively. The angular velocity of the Ch-LC droplets decreases monotonically with decreasing external temperature gradient, until they eventually stop rotating at a temperature gradient of d*T*/d*z* ~ 0 mK/µm, as shown in Fig. 1c. When we invert the temperature gradient applied to the Ch-LC droplets, they rotate in the reverse direction. This behavior is characteristic of the cross-correlation in the chiral system^27,28^.

To investigate the relationship between the droplet rotation and the temperature gradient magnitude, we measured the time evolution of the rotational angle of the droplets, θ, which is summarized in Fig. 1e. θ is proportional to time *t*, indicating that the angular velocity of the Ch-LC droplets, ω, is constant. We fit the experimental results with a linear function (the dashed line in Fig. 1e), and estimated ω from the slope of the linear function; the values of ω are (B) − 0.11 rad/s under − 4.5 mK/µm, (C) 0.01 rad/s under 0.04 mK/µm, and (D) 0.11 rad/s under 3.1 mK/µm.

We applied the uniform external temperature gradient to Ch-LC droplets. Heat flux is defined as the multiplication of the temperature gradient and heat conductivity of Ch-LC (~ 0.2 W/K m)^29^. The cross-correlation converts the heat flux along the temperature gradient into torque, which causes the Ch-LC droplets to rotate. The normalized angular momentum of the droplets is expressed as *R*^2^ω, where *R* is the radius of the Ch-LC droplet, which depends on the temperature gradient as

1 $$R^{2} \upomega = ({\text{d}}T/{\text{d}}z)/\upalpha_{{\text{L}}} ,$$

where α_L_ is the normalized parameter of the cross-correlation (Lehmann) coefficient and the viscosity constant. The effects of the viscosity and size appear in α_L_ and *R*^2^ω, respectively. The angular velocity depends on the viscosity of the Iso phase. We control the average temperature to be constant Ch-Iso coexisting temperature. Since the cell gap and the maximum temperature gradient in our setup are 40 μm and 6 mK/μm, respectively, the temperature deviation from average δ*T* is must be less than 0.24 K. The temperature dependence of viscosity of the Iso phase is weak, therefore is negligible and we can use average viscosity constant of η(*T*_C_) = {η(*T*_C_ + δ*T*) + η(*T*_C_ − δ*T*)}/2, where *T*c is the Ch-Iso coexisting temperature. Thus, we can regard α_L_ as the conversion efficiency from temperature gradient to angular momentum.

**Figure 2:**
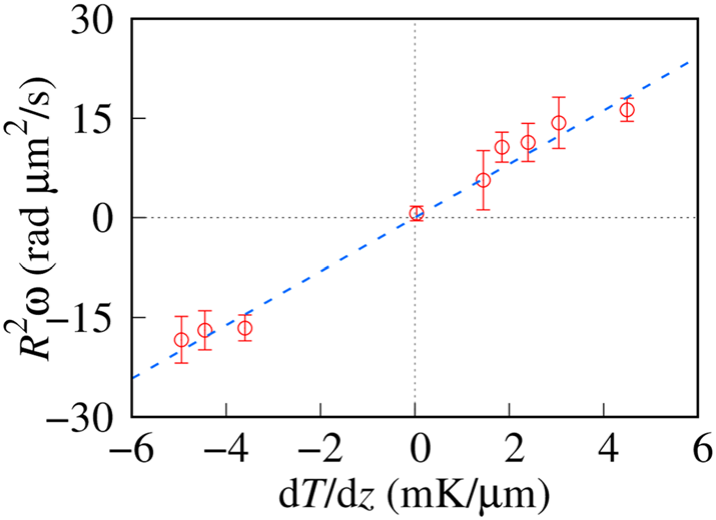
Temperature gradient dependence of the normalized angular momentum. The dashed line is the best-fit curve obtained using Eq. (1). The slope of the fitting function is 4.0 rad µm^3^/mK s. Each data point is averaged over more than 4 samples.

To estimate α_L_, we summarized *R*^2^ω as a function of d*T*/d*z*, as shown in Fig. 2. We fit the experimental results with a linear function, and the best-fit curve is shown as the dashed line in Fig. 2. *R*^2^ω is proportional to d*T*/d*z* within the range of our measurements (|d*T*/d*z*|< 6 mK/µm), resulting in a conversion efficiency, α_L_, of 0.25 ± 0.01 mK s rad^−1^ µm^−3^. α_L_ is the coupling constant between the temperature gradient and the angular momentum of the Ch-LC droplets and is therefore a key parameter for use in estimating the local temperature gradient extent.

### Temperature gradient distribution around an Au thin-film electrode

We heated Au thin-film locally by applying an electric current to the Au thin-film electrode patterned on the lower glass substrate. Temperature gradient arises and the resulting heat flux in steady-state, *q* is defined as the multiplication of d*T*/d*z* and heat conductivity of Ch-LCs. To obtain steady-state rotation of Ch-LC droplets, we apply a constant electric current for a long time (> 5 min) before the observation.

Then, we observed the rotational behavior of the Ch-LC droplets on the Au thin-film electrode. This study examined the distribution of micro-temperature gradient through Ch-LC droplets between the upper and lower glass substrates with a spacing of 40 µm. To investigate the effect of local temperature gradient from the Au thin-film electrode, we did not apply any external temperature gradient. In other words, we controlled the temperature using a homemade temperature controller so that the upper and lower glass substrates are the same and the average temperature is to be the Ch-Iso coexisting phase.

**Figure 3:**
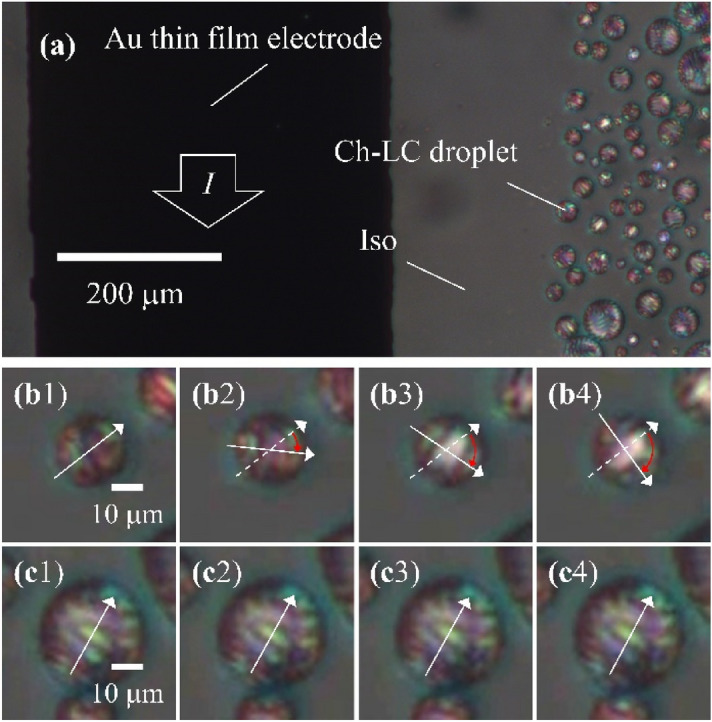
Polarized microscopic images of Ch-LC droplets forming on the glass substrate. We patterned the lower glass substrate with an Au thin-film electrode. (**a**) Au thin-film electrode (dark region) and Ch-LC droplets in the Iso phase. Also shown are polarized microscopic images of Ch-LC droplets at (**b**) *D* = 200 µm (Supplementary movie 4) and (**c**) *D* = 370 µm (Supplementary movie 5). Snapshots were collected every 10 s.

We applied 300 mA of current to the Au thin-film electrode and cooled the Ch-LC sample from the Iso phase. The Ch-LC droplets appear at the Ch-LC coexisting phase temperature. Figure 3a shows a transmission polarized microscopic image of Ch-LC droplets on the electrode. The dark region in Fig. 3a corresponds to the area containing the patterned Au thin-film electrode on the lower glass substrate; the electrode appears dark because it does not transmit light. Ch-LC droplets start to appear at some distance from the Au electrode with decreasing temperature, indicating that the temperature of the electrode increases and thus, the temperature gradient in the plane parallel to the glass substrates also increases. In this work, we focus on micro-temperature gradient detection perpendicular to the glass substrates rather than on the millimeter-scale in-plane gradient. However, understanding how the behavior of Ch-LC droplets develops should provide information about the in-plane temperature as well.

Figure 3b contains polarized microscopic images of a Ch-LC droplet at *D* = 200 µm, where *D* is the distance from the edge of the electrode to the center of the Ch-LC droplet (Supplementary movie 4). The Ch-LC droplet near the Au thin-film electrode rotates clockwise. As ω < 0 corresponds to d*T*/d*z* < 0, as shown in Fig. 2, this result suggests that Ch-LC droplets can detect upward temperature gradient.

Snapshots of Ch-LC droplets at *D* = 370 µm is shown in Fig. 3c and Supplementary movie 5. These Ch-LC droplets do not exhibit unidirectional rotation, indicating that the temperature gradient between glass substrates with a 40-µm spacing is too small to be detected by the Ch-LC droplets. These results suggest that the upward temperature gradient generated from Au thin-film electrodes decays with increasing *D*.

**Figure 4:**
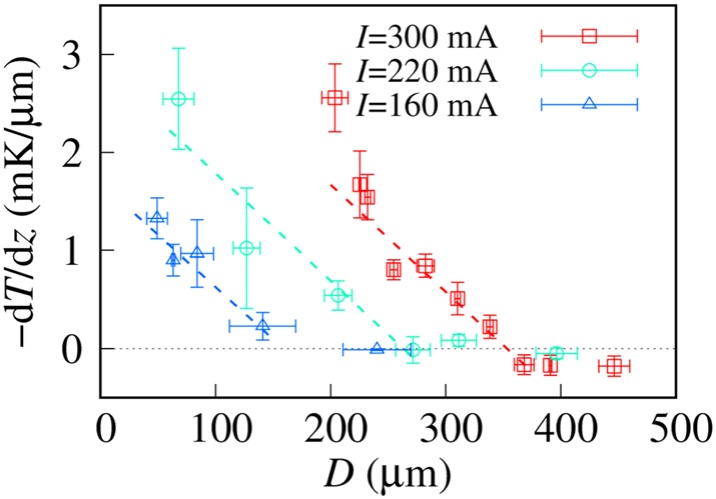
The distribution of upward temperature gradient, with local temperature gradient estimated from the angular momentum distribution of the Ch-LC droplets. The current values applied to the Au thin-film electrode are 300, 220, and 160 mA. The dashed lines are best-fit lines using linear functions. The slope is 0.01 ± 0.003 mK/µm^2^ and is independent of *I*. Each data point is averaged over more than 3 samples.

To quantify the temperature gradient distribution, we measured the values of *D* and *R*^2^ω for Ch-LC droplets from polarized microscopic images. As the temperature gradient amount is proportional to the normalized angular momentum^13,15^, substituting the experimental results into the following equation,

2 $$\frac{{{\text{d}}T}}{{{\text{d}}z}}(D) = \upalpha_{{\text{L}}} R^{2} \upomega (D),$$

gives the local temperature gradient distribution summarized in Fig. 4. An upward micro-temperature gradient was observed in the region *D* < 350 µm in the case of *I* = 300 mA, where *I* is the electric current. The temperature gradient decays proportionally to *D* and the amount of temperature gradient detected by the rotating Ch-LC droplets is a few mK/µm. To evaluate the detection accuracy of the Ch-LC droplets quantitatively, we fit the experimental results in the region of significant temperature gradient detection, which yielded a linear function (the dashed line in Fig. 4) with a slope of 0.01 ± 0.003 mK/µm^2^.

Next, we investigated the relationship between the upward temperature gradient and the applied current. The Ch-LC droplets at *D* < 260 µm continued to detect the upward temperature gradient at a lower value of applied current (*I* = 220 mA). The amount of upward temperature gradient from the Au electrode with *I* = 220 mA is less than that at a current of 330 mA. When we decreased the applied current to 160 mA, the detection distance at which Ch-LC droplets can detect upward temperature gradient decreased, along with a decrease in the amount of detected temperature gradient. We fitted the experimental results for current values of *I* = 220 mA and 160 mA with a linear function and found a slope of 0.01 mK/µm^2^ that is independent of the applied current.

**Figure 5:**
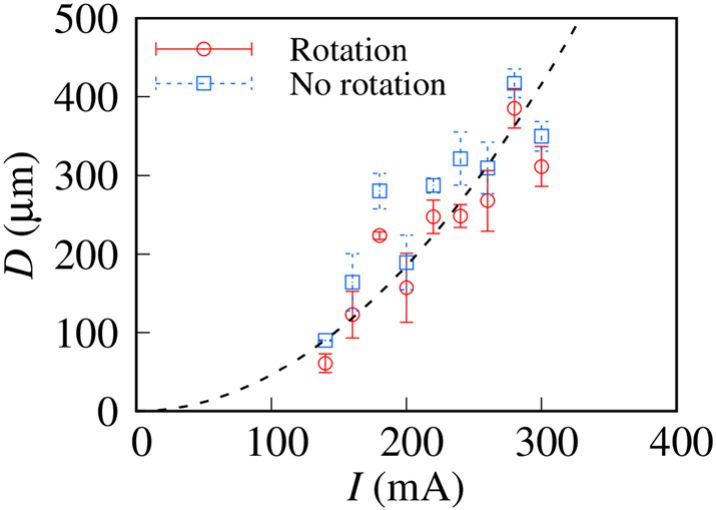
The phase diagram of rotational behavior concerning *I* and *D*. To distinguish between rotation and no rotation, we observed the rotational behavior of Ch-LC droplets for a long time (> 5 min). The boundary between regions of rotation and no rotation are indicated by a dashed line. Ch-LC droplets can detect micro-temperature gradient generated from the Au thin-film electrode in the region above the dashed line.

To better elucidate the region in which Ch-LC droplets can detect micro-temperature gradient, we investigated the *D* dependence of the droplet rotational behavior. The result is summarized in Fig. 5 as a phase diagram of the rotational behavior as a function of the applied current *I* and the position of the Ch-LC droplets, *D*. The fact that we could observe rotation of the Ch-LC droplets indicates that the upward temperature gradient is detectable, whereas a lack of rotation in the Ch-LC droplets indicates that the upward temperature gradient is smaller than the detection accuracy. When a large current is applied, the rotation region expands; the boundary between the regions of rotation and no rotation is proportional to *I*^2^ (the dashed line in Fig. 5), which is consistent with Joule heating behavior (∝*I*^2^).

## Discussion

### Detection resolution of temperature gradient distributions using Ch-LC droplets

The characteristic spatial scale of the micro-temperature gradient detection based on the rotation of Ch-LC droplets is the same as the droplet size (~ 10 µm). Using the cell gap (~ 40 µm) and the slope of the linear function in Fig. 4 (~ 0.01 ± 0.003 mK/µm^2^), we found that Ch-LC droplets can detect micro-temperature gradient with an accuracy of 0.4 ± 0.12 mK/µm. In addition, the microscopic field of view limits the region of temperature gradient detection. The field of view in our experimental system is ~ 1 mm^2^, which could be expanded by scanning the rotating Ch-LC droplets.

Temperature gradient detection using Seebeck elements must be accompanied by an increase in element size to increase the detection accuracy of the temperature gradient, which decreases the spatial resolution. On the other hand, smaller Ch-LC droplets rotate faster even under a constant temperature gradient^13^. Therefore, as the size of the Ch-LC droplets corresponds to the spatial resolution, such droplets represent a sensing system capable of maintaining both high detection accuracy and high spatial resolution.

### Numerical analysis of heat flux from Au thin-film electrodes

**Figure 6:**
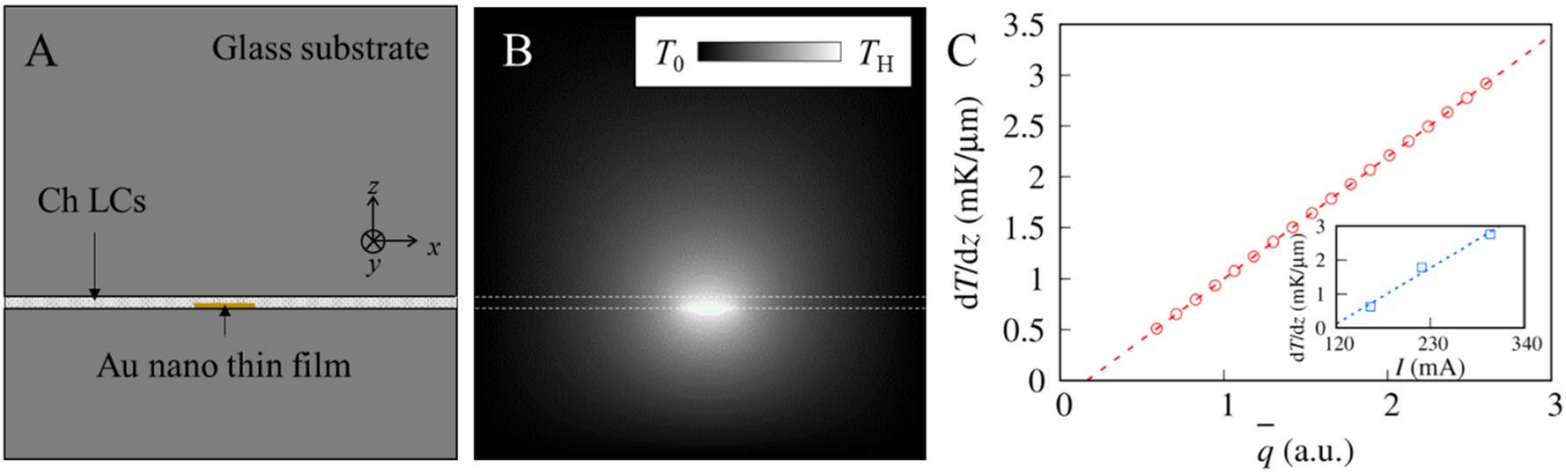
Numerical analysis of the upward micro-temperature gradient generated from Au thin-film electrodes. (**a**) A schematic cross section of the calculation area, which corresponds to the α-α′ cross section in Fig. 1(**a1**). The Ch-LCs are sandwiched between glass substrates. The temperature at far distances from the Au thin-film electrode was maintained at *T*_0_. (**b**) The temperature distribution obtained by numerical analysis with $\overline{q}$ = 0.5. Ch-LCs occupy the area between the white dashed lines. (**c**) $\overline{q}$-dependence of the temperature gradient at *D* = 100 µm. We normalized $\hat{q}$ as $\overline{q} = \hat{q}/\hat{q}(\nabla T = 1 \,{\text{mK}}/\upmu {\text{m}})$. The dashed line shows the fit of the numerical results using a linear function. The inset displays the *I* dependence of the experimental temperature gradient.

We calculated the heat flux from the Au thin-film electrodes based on the thermal conduction equation. Figure 6a contains a schematic image of the numerical analysis. The calculation area is given by the cross-section α-α′ shown in Fig. 1a. The thicknesses of the upper substrate, lower substrate, and Ch-LC are 2 mm, 1 mm, and 40 µm, respectively, which correspond to our experimental conditions. Here, we assumed that the heat conductivity of glass equals that of Ch-LC. The length of the Au thin-film electrode (> 10 mm) is sufficiently greater than both its width (~ 500 µm) and its thickness (~ 200 nm). Therefore, we could regard the temperature distribution along the *y*-axis as uniform, with the temperature gradient arising only in the *x*–*z* plane. The rotating Ch-LC droplets were observed after applying an electric current for a sufficient duration, so that the temperature distribution can be regarded as steady-state (∂*T*/∂*t* = 0). Heat is generated only by Au thin-film electrodes, that is, by the glass substrates, whereas the Ch-LC do not generate heat. We then solved the following thermal conduction equations using a finite-difference method to determine the temperature distribution in steady-state

3 $$0 = \left\{ {\begin{array}{*{20}l} {\left( {\frac{{\partial^{2} }}{{\partial \hat{x}^{2} }} + \frac{{\partial^{2} }}{{\partial \hat{z}^{2} }}} \right)T\quad ({\text{In}}\,{\text{LC}}\,{\text{or}}\,{\text{glass}}) } \hfill \\ {\left( {\frac{{\partial^{2} }}{{\partial \hat{x}^{2} }} + \frac{{\partial^{2} }}{{\partial \hat{z}^{2} }}} \right)T + \hat{q} \quad ({\text{In}}\,{\text{Au}}\,{\text{nano}}\,{\text{thin-film}})} \hfill \\ \end{array} } \right..$$

We used normalized parameters $\hat{x}$ = *x*/*L*, $\hat{y}$ = *y*/*L*, and $\hat{q}$ = $\dot{q}$/*kL*^2^, where *L*, *k*, and $\dot{q}$ are the width, thermal conductivity, and heat flux density of the Au thin-film electrode, respectively. The boundary condition was set so that the temperature far from the Au thin-film electrode was *T*_0_.

Figure 6b shows a typical temperature distribution obtained by our numerical calculation results. Ch-LCs occupy the region between the white dashed lines. The glass substrates lie outside the Ch-LCs. The temperature increases in the vicinity of the Au thin-film electrode, whereas the temperature is equal to *T*_0_ far from the Au thin-film electrode. The temperature difference between the upper and lower substrates relaxes on a length scale of 0.1–1 mm.

We compared the numerical and experimental results by calculating the temperature gradient distribution from the temperature distribution obtained by our numerical analysis. The results demonstrate that the temperature gradient at *D* = 100 µm, which is proportional to temperature gradient, is a function of the normalized heat $\overline{q}$, where we normalized ${\hat{\text{q}}}$ as $\overline{q} = \hat{q}/\hat{q}(\Delta T = 1 \,{\text{mK}}/\upmu {\text{m}})$. The temperature gradient observed at a fixed point is proportional to ${\overline{\text{q}}}$, as indicated by the dashed line in Fig. 6c.

In our experiment, we modulated the heat generation from the Au thin-film electrode by controlling the applied current *I*; $\stackrel{\mathrm{-}}{\text{q}}$ in the numerical analysis should be proportional to *I* in the experiment. The experimental temperature gradient at *D* = 100 µm is linearly dependent on *I*, as shown in the inset of Fig. 6c. Thus, our numerical analysis quantitatively agrees with the experimental results, indicating that Ch-LC droplets are capable of detecting upward micro-temperature gradient amounts generated from model MEMS devices.

## Materials and methods

### Chiral liquid crystal materials

To obtain the Ch-LCs used in this study, we added 1.0 wt.% of a chiral dopant, (*S*)-4-{[1-(methylheptyl)oxy]carbonyl}phenyl-4(hexyloxy) benzoate, to a host nematic LC E7. Then, we added 17 wt.% of octadecane (Sigma-Aldrich) to the Ch-LCs to expand the coexisting phase temperature of Ch-Iso. The coexisting phase temperature of the Ch-LCs is 33 °C–37 °C.

### Nano Au thin-film electrode

**Figure 7:**
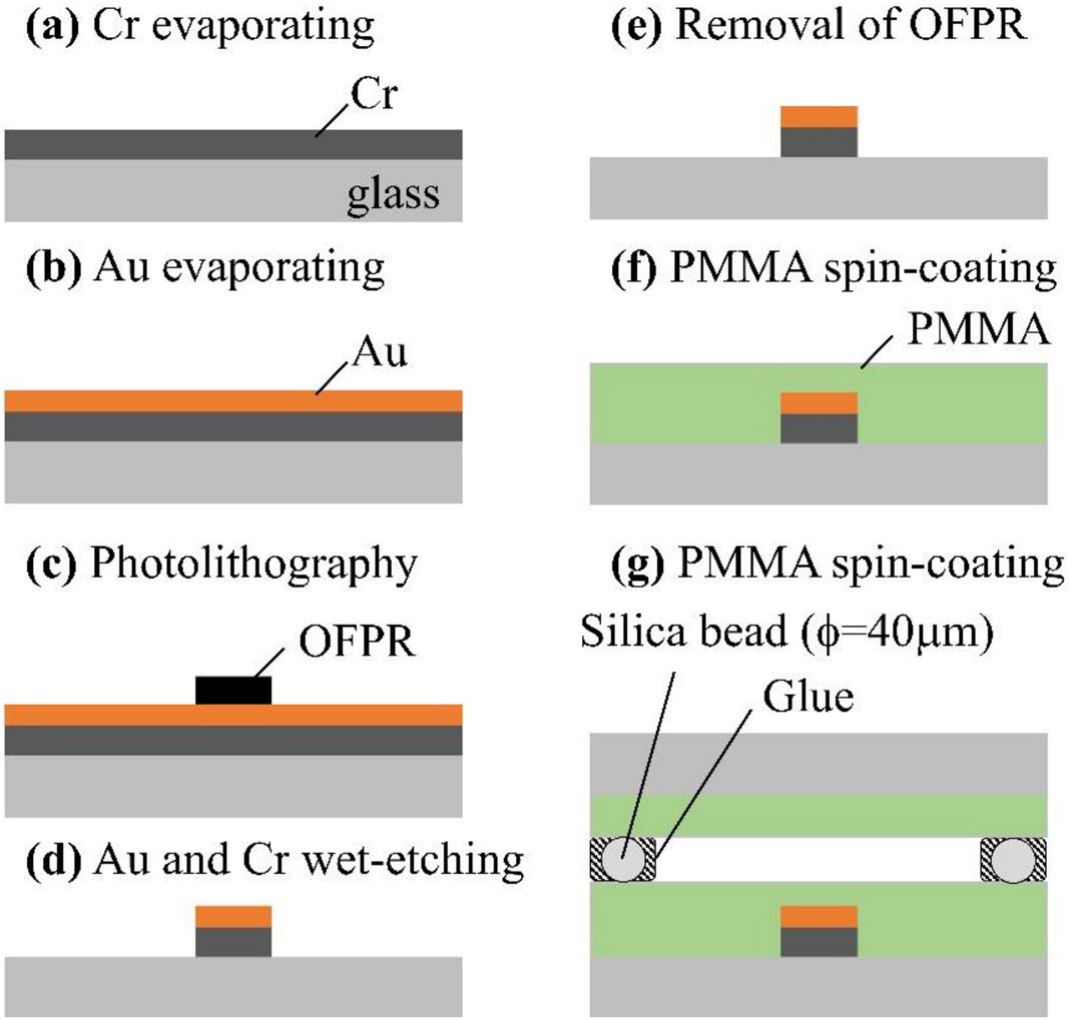
Schematic diagram of the model MEMS device fabrication process. (**a**,**b**) Thermal evaporation of Cr and Au layers. (**c**) Patterning of OFPR using a photolithography technique. (**d**) Wet-etching of the Au and Cr layers. (**e**) OFPR removal by washing with an organic solvent. (**f**) Spin coating of the PMMA alignment film. (**g**) LC cell assembly with a thickness maintained at 40 µm by bead spacers.

The fabrication process of the model MEMS device in which we patterned the glass substrate with an Au thin-film electrode is illustrated in Fig. 7. A 50-nm-thick Cr layer and a 200-nm-thick Au layer were deposited on the glass substrates by thermal evaporation (Fig. 7a,b). We obtained the OFPR pattern on the Au thin film using a photolithography process (Fig. 7c). During the wet-etching process, Au and Cr films were partially removed (Fig. 7d) and the OFPR was then washed out with acetone and ethanol (Fig. 7e). We spin-coated a toluene solution containing 2 wt.% PMMA to produce a PMMA alignment film after leaving the substrates at 150 °C for 120 min (Fig. 7f). We combined the PMMA-coated glass substrate with the substrate, using 40-µm-size silica beads (Fig. 7g). The width of the Au thin-film electrode is 440 µm.

### Microscopy and temperature controller

**Figure 8:**
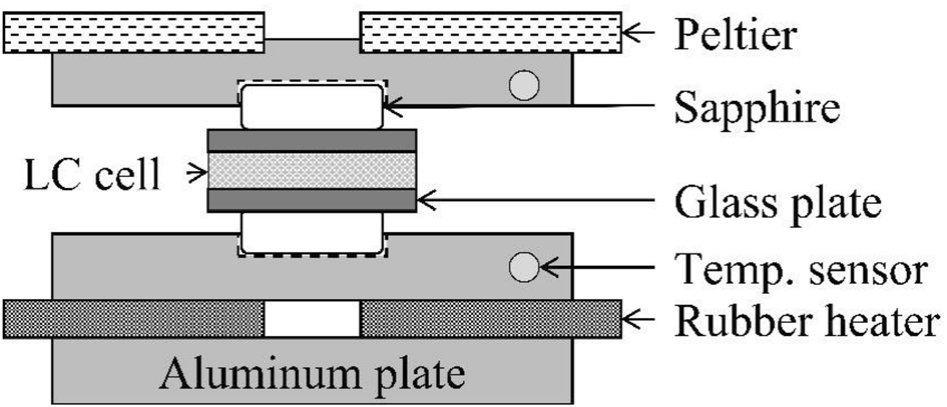
A schematic image of our constructed temperature controller. The LC cell is sandwiched between sapphire glass. The temperatures of the upper and lower aluminum plates are independently controlled by a Peltier element and a rubber heater, respectively.

We enclosed Ch-LCs into the fabricated LC cell. The LC cell was then set in the homemade temperature controller, as shown in Fig. 8. The lower and upper aluminum plates were heated by a rubber heater and a Peltier element, respectively. We monitored the temperatures of the upper and lower aluminum plates and could control the temperature within 0.1 °C. We connected the Au thin-film electrode with a DC power supply via 15 W of a resistor. We also conducted polarized microscopic observations of the Ch-LC droplets using a cross-Nicol polarized at 10° away to simultaneously observe both the texture of the Ch-LC droplets and the Ch-Iso interface.

## Conclusion

In this paper, we demonstrated a novel application of rotating Ch-LC droplets to detect micro-levels of temperature gradient generated by model MEMS devices. First, we evaluated the temperature gradient-to-torque conversion efficiency of Ch-LC droplets under conditions of uniform temperature gradient. Then, we patterned a glass substrate with an Au thin-film electrode and fabricated model MEMS devices using Ch-LC droplets. We then investigated the detection of spatially heterogeneous temperature gradients based on the rotational behavior of Ch-LC droplets. Ch-LC droplets were able to detect tiny quantities of temperature gradient (~ 1 mK/μm) generated by the model MEMS device. In this novel method, we demonstrate that the Ch-LC droplets exhibit both high accuracy (~ 0.4 ± 0.12 mK/µm) and high spatial resolution (~ 1–10 µm) for temperature gradient detection.

## Supplementary Information

Supplementary Video 1.

Supplementary Video 2.

Supplementary Video 3.

Supplementary Video 4.

Supplementary Video 5.

Supplementary Legends.

## Data Availability

The data that support the findings of this study are available from the corresponding author upon reasonable request.
