# Supplementary material for: Temperature gradient sensing mechanism using liquid crystal droplets with 0.1-mK-level detection accuracy and high spatial resolution
Source: Sci Rep. 2022 Aug 12;12:13733. doi: 10.1038/s41598-022-18008-y (PMC9374739; doi:10.1038/s41598-022-18008-y)
Supplement: Supplementary file 6 — Supplementary Legends. [file 41598_2022_18008_MOESM6_ESM.docx]

Supplementary movies for

**Heat flux sensors using chiral liquid crystal droplets with 0.1-mK-level detection accuracy and high spatial resolution**

Shinji Bono, Satoshi Konishi

*Corresponding author. Email: [bono@fc.ritsumei.ac.jp](mailto:bono@fc.ritsumei.ac.jp)

Supplementary movie 1.

Movie corresponding to Fig. 1b Ch-LC droplets under uniform heat flux (∇*T* = −4.5 mK/μm). The recording was performed with 6 times faster speed.

**Supplementary movie 2.**

Movie corresponding to Fig. 1c. Ch-LC droplets under uniform heat flux (∇*T* = −0.04 mK/μm). The recording was performed with 6 times faster speed.

**Supplementary movie 3.**

Movie corresponding to Fig. 1d. Ch-LC droplets under uniform heat flux (∇*T* = 3.1 mK/μm). The recording was performed with 6 times faster speed.

**Supplementary movie 4.**

Movie corresponding to Fig. 3b. A Ch-LC droplet at *D* = 200 μm. The recording was performed with 6 times faster speed.

**Supplementary movie 5.**

Movie corresponding to Fig. 3c. A Ch-LC droplet at *D* = 370 μm. The recording was performed with 6 times faster speed.
